# Combination therapy with ipilimumab and electrochemotherapy: preliminary efficacy results and correlation with immunological parameters

**DOI:** 10.1186/1479-5876-12-S1-O5

**Published:** 2014-05-06

**Authors:** Ester Simeone, Lucia Benedetto, Giusy Gentilcore, Marilena Capone, Corrado Caracò, Gianluca Di Monta, Ugo Marone, Marialuisa Di Cecilia, Antonio Maria Grimaldi, Stefano Mori, Nicola Mozzillo, Paolo A Ascierto

**Affiliations:** 1 Istituto Nazionale Tumori, Fondazione “G. Pascale”, Naples, Italy

## Background

Ipilimumab is the first agent approved for the treatment of advanced melanoma that showed a survival benefit in randomized phase 3 trials. Despite the survival benefit, due to its mechanism of action it is associated with a slow onset and low rate of responses and, in many cases, responses occur after other therapies, like chemotherapy, targeted therapy and radiotherapy. Electrochemotherapy (ECT) has been shown to be effective and well tolerated for local control of metastatic melanoma with superficial lesions. The current challenge is to improve ipilimumab efficacy by combination/sequence with other therapies. We performed a pilot study of combination with ipilimumab and ECT in order to verify the possible increase of response rate. Furthermore, due to the lack of predictive markers, we evaluated the possible predictive role of circulating T-regulatory cells (T-Reg) variations in peripheral blood mononuclear cells (PBMC) of treated patients.

## Methods

We collected data from 15 patients (pts) with advanced melanoma (9 stage IIIc and 6 stage IV M1c) treated with ipilimumab at 3 mg/kg every 3 weeks for 4 cycles (day 1) and ECT with bleomycine at 15 mg/m2 (day 2) on superficial lesions. Blood draws were collected on day 0, 1 and 2, then on day 15 and 30 from ECT, at each cycle of ipilimumab and at every tumor evaluation (every 12 weeks). PBMC were thawed and labeled with anti-CD4-Pe-Cy-5, CD25-Pe and anti-FoxP3-AlexaFlour488 for T-Reg.

## Results

10/15 (67%) pts showed local objective responses (4 CR and 6 PR). 7/10 (70%) pts showed local response (6 PR and 1 CR) after the second ipilimumab dose and 3/10 showed response (3 CR) at week 12. Two pts with PR (28%) out of the group of 7 showed response on distant lesions at week 24 (abscopal effect). All pts are still alive with a median follow up of 12.4 months (range 6-18).

We found in all responders a decrease of T-Reg of 0.10% (range 0.50-2.6%) per cycle and no variation of CD4+ and CD25+ lymphocytes. Furthermore 5/15 pts not responders had a delayed stable disease (local and distant) at week 16, with no variations of T-Regs.

## Conclusion

Our preliminary results show that a combination approach with ipilimumab and ECT may increase responses to ipilimumab. T-Reg decrease in PBMC could be associated with early response to treatment. Further studies about this combination are warranted.

